# Endoscopic Stenting for Unresectable Malignant Hilar Biliary Obstruction: Where Do We Stand Today? A Narrative Review

**DOI:** 10.3390/curroncol32110608

**Published:** 2025-10-31

**Authors:** Tadahisa Inoue, Itaru Naitoh, Michihiro Yoshida, Fumihiro Okumura

**Affiliations:** 1 Department of Gastroenterology, Aichi Medical University, 1-1 Yazakokarimata, Nagakute 480-1195, Aichi, Japan; 2 Department of Gastroenterology, Nagoya City University Midori Municipal Hospital, 1-77 Shiomigaoka, Midori-ku, Nagoya 458-0037, Aichi, Japan; inaito@med.nagoya-cu.ac.jp; 3 Department of Gastroenterology and Metabolism, Nagoya City University Graduate School of Medical Sciences, 1 Kawasumi, Mizuho-cho, Mizuho-ku, Nagoya 467-8601, Aichi, Japan; mityoshi@med.nagoya-cu.ac.jp; 4 Department of Gastroenterology, Gifu Prefectural Tajimi Hospital, 5-161 Maehata-cho, Tajimi 507-8522, Gifu, Japan; okumura-fumihiro@tajimi-hospital.jp

**Keywords:** malignant hilar biliary obstruction, plastic stent, metal stent

## Abstract

**Simple Summary:**

Blockage of the bile ducts at the point where they meet inside the liver can occur in patients with advanced cancers of the bile duct, gallbladder, pancreas, or liver. This condition causes yellowing of the skin, itching, and infection, and it requires drainage of bile to relieve symptoms and improve liver function. When surgery is not possible, endoscopic placement of small tubes called stents can reopen the blocked ducts. Many different stent types and techniques have been developed, but the best approach is still debated because the anatomy in this region is very complex. This review summarizes the current knowledge about how to select the best drainage strategy, including which part of the liver should be drained and which stent type is most effective. It also describes new techniques such as drainage guided by ultrasound from inside the body. The findings highlight the need for treatments that not only last longer and are easier to repeat but also improve comfort and quality of life for people living with advanced disease. The review aims to help doctors choose the safest and most effective treatment for each patient.

**Abstract:**

Malignant hilar biliary obstruction (MHBO) is a complex clinical condition commonly observed in individuals with advanced cholangiocarcinoma and other hepatobiliary malignancies. Endoscopic stenting remains the primary palliative intervention for unresectable cases; however, the optimal strategy has not been clearly defined owing to the anatomical intricacies of the hilar region and the heterogeneity of disease presentation. This narrative review summarizes current evidence and ongoing debates on functional liver volume-based drainage, unilateral versus bilateral stenting, stent type selection, and above-the-papilla approaches. In addition, we highlight recent progress in endoscopic ultrasound-guided biliary drainage as a promising alternative or complementary approach when conventional transpapillary drainage proves inadequate. With recent advances in systemic therapies, including immunotherapy, patient survival has improved, underscoring the need for durable, reintervention-friendly stent strategies. Although uncovered metal stents have long been regarded as the standard for unresectable cases, their limitations in reintervention have prompted renewed consideration of both stent type and drainage technique. Robust prospective studies remain essential to establish standardized, evidence-based guidelines that optimize patient benefit and improve long-term outcomes in unresectable MHBO.

## 1. Introduction

Malignant hilar biliary obstruction (MHBO) arises from various primary and secondary malignancies, including cholangiocarcinoma, gallbladder cancer, pancreatic cancer, liver metastasis, peritoneal dissemination, and lymph node metastasis. Obstruction at the hilar confluence results in obstructive jaundice and/or cholangitis, necessitating prompt and effective biliary drainage. Biliary drainage not only alleviates symptoms but also improves hepatic function, enables subsequent systemic therapy, and ultimately enhances both prognosis and quality of life.

Among available modalities, endoscopic biliary stenting has become the preferred approach for unresectable MHBO because of its minimally invasive nature, reproducibility, and relatively favorable safety profile compared with percutaneous or surgical interventions [1,2]. However, stent placement in the hilar region is technically demanding because of the complex biliary anatomy, characterized by multiple branches, sharp angulations, and variable ductal confluence. The clinical presentation of MHBO is highly heterogeneous depending on the site, extent, and anatomical configuration of the stricture.

The management of MHBO presents greater technical and strategic challenges than malignant distal biliary obstruction. Successful drainage requires meticulous preprocedural planning, including identification of the functional liver volume to be drained, determination of unilateral versus bilateral drainage, and appropriate stent type (plastic stent [PS] versus metal stent [MS]; covered versus uncovered designs).

Despite significant advances over the past 2 decades, no universal consensus has been reached on the optimal drainage strategy for unresectable MHBO. Innovations in device design, imaging modalities, and alternative techniques such as endoscopic ultrasound-guided biliary drainage (EUS-BD) have expanded therapeutic possibilities, yet many questions remain unresolved. A comprehensive understanding of the existing evidence base is therefore essential to maximize patient benefit and guide individualized treatment strategies.

In this narrative review, we summarize the current evidence, clinical practice considerations, and ongoing challenges of endoscopic stenting for unresectable MHBO, highlighting key controversies and emerging approaches that are likely to influence future practice. This narrative review was conducted based on a comprehensive literature search using the PubMed and Google Scholar databases for studies published up to August 2025. The search terms included “malignant hilar biliary obstruction,” “malignant hilar biliary stricture,” “endoscopic stenting,” “endoscopic drainage,” “metal stent,” “plastic stent,” and “EUS-guided biliary drainage.” Both randomized controlled trials and high-quality observational studies published in English were included. The reference lists of key articles and relevant review papers were also screened to ensure completeness. Given the narrative nature of this review, no formal quality assessment or meta-analysis was performed.

## 2. Functional Liver Volume to Be Drained

In MHBO, the left and right intrahepatic ducts may become functionally separated depending on the location and extent of the stricture, which is traditionally categorized by the Bismuth classification (types I–IV) according to proximal ductal involvement. This anatomical and physiological partitioning necessitates an early strategic decision: whether to deploy a single stent to one hepatic system (unilateral drainage) or to pursue bilateral drainage with multiple stents. Although complete drainage of all obstructed segments is physiologically ideal, bilateral stenting is technically more demanding, may increase adverse events, and can complicate reinterventions after stent occlusion. Additionally, adequate clinical decompression can be achieved with unilateral drainage in selected patients. Accordingly, the critical consideration is not only whether sufficient bilirubin reduction can be achieved, but also how stent configuration influences longer-term outcomes such as stent patency and overall survival.

Three randomized controlled trials (RCTs) [3,4,5] have compared unilateral and bilateral strategies in unresectable MHBO. In an early RCT using PS, De Palma et al. [3] reported higher technical success (88.6% vs. 76.9%, *p* = 0.041) and fewer complications (18.9% vs. 26.9%, *p* = 0.026) with unilateral placement; however, this 2001 study predates modern devices and techniques and should therefore be interpreted with caution. More recently, Hakuta et al. [4] randomized patients to unilateral or bilateral endoscopic nasobiliary drainage followed by uncovered MS. Functional success did not differ (57% vs. 56%, *p* = 0.99), but additional drainage was required more frequently after unilateral therapy, while time to recurrent biliary obstruction and overall survival were comparable (*p* = 0.11 and 0.78, respectively). Notably, the number of patients who ultimately received MSs was relatively small (19 unilateral vs. 25 bilateral). In a multicenter RCT using uncovered MSs, Lee et al. [5] reported similar technical success (95.5% vs. 100%, *p* = 0.244) but higher clinical success (95.3% vs. 84.9%, *p* = 0.047) and longer cumulative stent patency (median 252 days vs. 139 days, *p* < 0.01) with bilateral placement. Bilateral drainage also emerged as an independent favorable factor for patency (hazard ratio 0.30, 95% confidence interval [CI] 0.172–0.521, *p* < 0.001) and survival (hazard ratio 0.415, 95% CI 0.259–0.666, *p* < 0.01). Collectively, these trials highlight a balance between procedural complexity and early adverse events versus long-term patency and survival, with bilateral MS generally favored when technically feasible.

An updated systematic review and meta-analysis published in 2025 [6] synthesized the three RCTs together with numerous observational studies. Bilateral drainage achieved higher clinical success (risk ratio 0.97, 95% confidence interval 0.94–0.99, *p* = 0.01) with similar technical success (risk ratio 1.01, 95% confidence interval 1.00–1.03, *p* = 0.09) compared with unilateral drainage; however, early adverse events were more frequent with bilateral placement (risk ratio 0.59, 95% confidence interval 0.45–0.77, *p* = 0.0001), and late adverse event rates were similar (risk ratio 0.87, 95% confidence interval 0.74–1.03, *p* = 0.11). Importantly, bilateral stenting was associated with improved stent patency (hazard ratio 0.76, 95% confidence interval 0.66–0.87, *p* = 0.0001) and overall survival (hazard ratio 0.77, 95% confidence interval 0.68–0.87, *p* < 0.0001), with subgroup analyses favoring endoscopic bilateral metal stenting. These data support bilateral MS as the default strategy when patient anatomy and operator expertise allow, while acknowledging the early event penalty.

Beyond a binary “unilateral vs. bilateral” framing, several studies emphasize how much liver volume is drained as the determinant of success. Vienne et al. [7] first demonstrated that draining more than 50% of total liver volume predicted effective drainage and longer survival (119 days vs. 59 days, *p* = 0.005), often necessitating bilateral stenting. Takahashi et al. [8] refined this by CT volumetry (odds ratio 2.92, 95% confidence interval 1.648–5.197, *p* < 0.001), showing thresholds of ≥33% for patients with preserved liver function (normal liver or compensated cirrhosis) and ≥50% for those with impaired function (decompensated cirrhosis). They also cautioned that stenting an atrophic lobe predisposes patients to drainage-associated cholangitis. More recently, Morimoto et al. [9] used a three-dimensional image-based volume analyzer and found that achieving more than 80% drainage in patients receiving systemic chemotherapy was associated with significantly longer survival (median 450 days vs. 224 days, *p* = 0.0033). On multivariable analysis, more than 80% drainage independently predicted overall survival (hazard ratio 0.35, 95% confidence interval 0.20–0.62, *p* = 0.0003). These findings suggest that a volume-targeted approach, prioritizing viable, non-atrophic segments, may better align drainage strategy with patient prognosis than a simple unilateral/bilateral dichotomy.

Putting these aspects together, for unresectable MHBO, especially when longer-term outcomes are important because patients are candidates for systemic therapy, a bilateral stenting strategy is generally favored if it achieves sufficient functional drainage volume and can be performed safely by experienced operators. At the same time, indiscriminate stenting of atrophic lobes should be avoided because of cholangitis risk, and individualized planning using cross-sectional imaging or volumetry is recommended to target at least 50% drainage (or higher, for example, more than 80% in systemic-therapy candidates) of viable liver parenchyma. Prospective studies are still needed to define precise thresholds and to integrate volume-based planning into routine practice, but emerging evidence supports a shift from “laterality-based” to functional-volume-based drainage strategies.

## 3. Stent Selection

Once the target drainage area has been determined, the next critical step is selecting the appropriate type of stent. Stents for MHBO are broadly classified into PS, uncovered MS, and covered MS. Each option carries distinct advantages and limitations, and the choice should balance technical feasibility, anticipated stent patency, reintervention strategy, and patient prognosis (Table 1).

### 3.1. Uncovered Metal Stent

Two RCTs [10,11] have directly compared uncovered MS with PS in a large cohort of patients with unresectable MHBO. Mukai et al. [10] reported that uncovered MS provided significantly longer median stent patency (359 vs. 112 days, *p* = 0.0002) and required fewer reinterventions (mean 0.63 vs. 1.80, *p* = 0.0008). Importantly, the overall cost of biliary drainage was lower in the uncovered MS group (*p* = 0.0222), reflecting the reduction in repeated procedures. Similarly, Sangchan et al. [11] demonstrated higher successful drainage rates with uncovered MS (70.4% vs. 46.3%, *p* = 0.002), as well as superior median stent patency (103 vs. 35 days, *p* < 0.001) and overall survival (126 vs. 49 days, *p* = 0.002). These data established uncovered MS as more effective and cost-efficient than PS, although both RCTs primarily involved unilateral stent placement.

Expanding on this, Xia et al. [12] conducted a large retrospective comparison of bilateral stenting strategies. Bilateral uncovered MS significantly outperformed bilateral PS in terms of clinical success (99.0% vs. 71.9%, *p* < 0.001), median symptom-free stent patency (9.2 vs. 4.8 months, *p* < 0.001), number of interventions (1.3 vs. 2.0, *p* < 0.001), and median survival (7.2 vs. 4.1 months, *p* = 0.015). These findings suggest that the superiority of uncovered MS over PS extends beyond unilateral drainage to bilateral configurations. For bilateral uncovered MS placement, two deployment techniques are available: stent-by-stent (SBS) and stent-in-stent (SIS). An RCT by Lee et al. [13] compared these approaches and found no significant differences in adverse event rates, technical success, clinical success, stent patency duration, or survival probability, indicating that both methods are reasonable options depending on operator expertise and anatomical considerations.

Taken together, uncovered MSs consistently demonstrate higher clinical success rates, longer stent patency, fewer interventions, lower overall costs, and improved survival compared with PS, with benefits especially pronounced in bilateral stenting. Accordingly, uncovered MSs have long been regarded as the standard of care for unresectable MHBO, particularly in patients with an expected survival of at least 3 months [1,14]. International guidelines and expert consensus statements continue to recommend uncovered MSs as first-line devices in this setting. Nevertheless, enthusiasm for uncovered MSs has recently been tempered by challenges surrounding reintervention after occlusion. As discussed in later sections, these limitations have prompted reconsideration of optimal stent selection and technique.

### 3.2. Plastic Stent

Although uncovered MSs have demonstrated superiority over PSs in terms of patency, reintervention remains a major limitation of uncovered MSs. Their primary mode of occlusion is tumor ingrowth, and once deployed, uncovered MSs are essentially non-removable. This makes reintervention for occluded stents technically complex, particularly after bilateral placement, where success rates are reported at only 76.3–85.7% [15,16,17,18,19]. With the ongoing improvement of antitumor therapies, including immune checkpoint inhibitors such as durvalumab and pembrolizumab, patients with MHBO, especially those with cholangiocarcinoma, are experiencing longer survival [20,21]. Consequently, even with bilateral uncovered MSs, approximately half of patients live long enough to develop stent occlusion [22]. Moreover, effective tumor shrinkage with systemic therapy has occasionally allowed conversion to surgical resection, further highlighting the importance of removable stents in selected cases. These factors have prompted renewed interest in PS, whose ease of removal greatly simplifies reintervention strategies.

The major drawback of PS remains its short patency period, typically necessitating frequent exchanges and reinterventions. To overcome this limitation, strategies to extend PS patency have been explored. One promising approach is above-the-papilla placement (also referred to as suprapapillary placement), in which the stent is positioned entirely within the bile duct without traversing the papilla, thereby reducing duodenobiliary reflux, which is a major contributor to stent occlusion [23,24]. In some countries, dedicated PS systems equipped with retrieval threads at the distal edge have been developed, allowing intentional intraductal placement and facilitating removal by pulling the thread during reintervention [25].

Two RCTs have evaluated this technique in unresectable MHBO. Kurita et al. [23] demonstrated that median cumulative stent patency was significantly longer with above-the-papilla PS compared with conventional across-the-papilla placement (123 vs. 51 days, *p* = 0.031), while technical success, clinical success, reintervention rates, adverse events, and survival were comparable. Kanno et al. [24] compared above-the-papilla PS placement with uncovered MS placement and found no significant differences in technical success (100% vs. 96.6%), clinical success (90.0% vs. 88.9%), adverse event rate (10.5% vs. 15.2%), or time to recurrent biliary obstruction (250 vs. 361 days, *p* = 0.34). These findings suggest that above-the-papilla PS can achieve outcomes similar to that via uncovered MSs while preserving the key advantage of removability.

In the current clinical era, characterized by prolonged patient survival, frequent need for reintervention, and the possibility of conversion surgery, PS may represent a viable first-line alternative to uncovered MSs in many cases of unresectable MHBO. Above-the-papilla PS placement, in particular, appears to extend patency and reduce reflux-related occlusion, positioning it as a potential option. However, evidence remains limited, and further prospective trials are needed to validate its role. Additionally, the limited availability of dedicated PS devices with retrieval mechanisms may restrict widespread adoption.

### 3.3. Covered Metal Stent

Given the limited patency of PSs, alternative strategies have increasingly gained attention, among which covered MSs are a key option. These stents were originally designed to prevent tumor ingrowth, a major limitation of uncovered MSs, while preserving the advantage of removability, a feature shared with PSs. Covered MSs are widely used for malignant distal biliary obstruction, but their application in MHBO has traditionally been limited because of concerns about occluding intrahepatic side branches, which can lead to segmental cholangitis. Recently, however, the introduction of slim-covered MSs with a 6 mm diameter, thinner than the conventional 8 mm or 10 mm MSs, has provided a promising alternative [26]. These devices are designed to minimize side-branch occlusion, maintain removability, simplify reintervention, and potentially achieve longer patency than PSs in the hilar setting.

The first RCT of slim-covered MS in unresectable MHBO was conducted by Paik et al. [27], comparing bilateral slim-covered MS with PS placement. The mean time to recurrent biliary obstruction was significantly longer in the slim-covered MS group (190 vs. 96 days, *p* = 0.02). Importantly, the rates of adverse events, including segmental cholangitis, were comparable between groups, although stent migration occurred more frequently with slim-covered MS. Notably, all stents in this trial were placed across the papilla, which may have influenced patency outcomes. Supporting evidence from four retrospective studies [26,28,29,30] suggests that above-the-papilla placement of slim-covered MS achieves a longer patency period, similar to that of PS. Consistent with findings in malignant distal biliary obstruction, where an RCT has shown superior patency of covered MS placed above the papilla compared with that across the papilla [31], the same trend appears to hold in hilar disease. A recent retrospective study [32] reported in 2025 compared above-the-papilla SBS bilateral placement of slim-covered MS with uncovered MS. The slim-covered MS demonstrated significantly longer time to recurrent biliary obstruction (median, not achieved vs. 204 days; *p* = 0.048), shorter reintervention procedure time (median, 20 vs. 31 min; *p* = 0.005), and fewer reinterventions (median, 1 [range, 1–2] vs. 2 [range, 1–6]; *p* = 0.029), indicating a potentially important role for slim-covered MS in the management of unresectable MHBO.

An important consideration is the distinction between fully covered and partially covered MSs. In reintervention studies using slim-covered MSs, the removability rate was 100% for fully covered stents but only 46–67% for partially covered designs [26,28,29,30]. Therefore, when removability is an important therapeutic goal, fully covered designs should be preferred over partially covered designs.

Based on the currently available evidence, slim-covered MS may represent a potential option for unresectable MHBO, particularly when placed above the papilla. However, most of the available data on slim-covered MS are derived from small retrospective series or early-phase trials. These findings should therefore be considered preliminary, and their applicability to broader clinical practice remains to be validated through further research. Prospective, multicenter studies are required to confirm the role of slim-covered MS in MHBO, clarify the benefits of above-the-papilla placement compared with PS, and directly compare their outcomes, reintervention rates, and survival with those of uncovered MS. At present, slim-covered MS should be regarded as an emerging but investigational option, best applied in experienced centers or within clinical trials, while awaiting more robust evidence.

## 4. Emerging Alternative Option: EUS-Guided Approach

Thus far, we have focused on the transpapillary approach using ERCP. However, given the technical complexity and limitations of conventional strategies, further alternative methods have been explored. With the advancement of EUS-guided techniques [33], EUS-BD has emerged as a potential option for managing unresectable MHBO [34].

Several approaches for bilateral drainage using EUS-BD have been reported: (1) combination of hepaticogastrostomy and hepaticoduodenostomy [35], (2) bridging to the right hepatic duct combined with hepaticogastrostomy [36,37], and (3) the SIS method using bridging and antegrade stenting [38,39]. Among these, approach (2) is technically simpler than approach (1) because it requires only a single puncture site and is more broadly applicable. Consequently, it is currently the most frequently reported method for bilateral drainage using EUS-BD [34,40]. As noted earlier, bilateral stenting remains the standard approach for unresectable MHBO. However, reintervention after bilateral uncovered MS placement can be technically demanding. In method (2), bridging is usually achieved with an uncovered MS, but because only a single access route is required, the difficulty of reintervention may be reduced. However, this advantage is less evident in complex cases such as Bismuth type IIIa or IV, where separate drainage of the right anterior and posterior branches is required. Moreover, selecting the contralateral bile duct during the bridging process is a particularly challenging step. Difficulties are especially likely when the branching angles between the right and left hepatic ducts are steep or when the stricture is long. In such cases, even if initial intervention is successful, reintervention may still be problematic. Similarly, although method (1) may simplify reintervention by providing two independent routes, the requirement for dual puncture sites may offset this advantage.

Another potential strategy is the combination of EUS-BD with conventional transpapillary drainage [41]. In this hybrid approach, transpapillary stenting is typically used to drain the right hepatic system, while the left hepatic system is managed with EUS-BD. Although this approach requires two access routes, it reduces the number of stents required per route, potentially simplifying future reinterventions compared with complex bilateral transpapillary stenting. Compared with bilateral drainage achieved solely by EUS-BD, this approach also has the advantage of avoiding the technically demanding bridging process described above.

At present, reports of EUS-BD for MHBO remain limited, and its use has mainly been described in cases where ERCP is technically unsuccessful or difficult. Nonetheless, EUS-BD is increasingly considered not only as a rescue strategy but also as a possible first-line option. In particular, combination approaches may help overcome the inherent limitations of conventional bilateral transpapillary stenting by providing independent access to the right and left hepatic ducts. However, EUS-BD for MHBO is still in an early exploratory stage. Given that the current evidence for EUS-BD in MHBO is limited to small observational and feasibility studies, these approaches should be regarded as exploratory. Further well-designed, prospective trials are required before their routine use can be recommended, to better clarify their efficacy, safety, and role in relation to established transpapillary approaches.

## 5. Conclusions and Future Perspectives

This review summarizes the current status and ongoing challenges of endoscopic stenting for unresectable MHBO. Because of the complex hilar anatomy and the multiple factors that influence drainage strategies, no universally optimal approach has yet been defined. In addition, recent advances in systemic antitumor therapies have reshaped the clinical landscape, underscoring the need for evidence that reflects current treatment contexts.

It should be acknowledged that most of the available evidence is derived from retrospective studies and single-center experiences, with considerable heterogeneity in patient populations, stent types, and procedural techniques. Randomized controlled trials remain limited in number and size, and potential publication bias cannot be excluded. Therefore, the conclusions of this narrative review should be interpreted with caution, and further multicenter prospective studies are warranted to establish standardized, evidence-based strategies.

In addition to technical and survival outcomes, future research should place greater emphasis on patient-reported outcomes, including quality of life, symptom relief, and functional recovery. As endoscopic drainage for unresectable MHBO is fundamentally a palliative intervention, optimizing patient comfort and minimizing procedure-related burden are crucial. Standardized tools to assess symptom control and patient satisfaction should be integrated into future prospective studies to ensure that therapeutic strategies align with patient-centered goals of care.

Economic considerations also play an essential role in decision-making for patients with unresectable MHBO, particularly in palliative settings. While uncovered MSs have higher upfront costs compared with plastic stents, they are generally associated with fewer reinterventions and lower overall treatment expenses in the long term. However, data on cost-effectiveness remain limited, and further studies incorporating economic analyses are warranted to guide optimal allocation of healthcare resources.

Future prospective studies should incorporate univariate and multivariate analyses to identify independent prognostic factors, such as patient age, tumor stage, hepatic volume, and liver functional reserve, that influence clinical success, stent patency, and survival outcomes. Such analyses will be essential to develop personalized drainage strategies for unresectable MHBO.

At the same time, novel devices, techniques, and combined approaches, such as volume-based strategies, the above-the-papilla concept, slim-covered stents, and EUS-guided drainage, are rapidly emerging. Continued innovation and rigorously designed prospective studies are essential to identify effective methods that can maximize patient benefit and improve long-term outcomes.

## Figures and Tables

**Table 1:** Comparison of characteristics of different stent types used for unresectable malignant hilar biliary obstruction.

Feature	Across-the-Papilla PS	Above-the-Papilla PS	Uncovered MS	Slim-Covered MS
Patency duration	Short (≈1–3 months)	Moderate (≈4–8 months)	Long (≈6–12 months)	Moderate–Long (≈6–10 months)
Removability	Easy (fully removable)	Easy (removable)	Not removable	Removable (especially fully covered)
Reintervention complexity	Simple	Simple	Difficult, especially after bilateral placement	Easier than uncovered MS if removable
Risk of ingrowth	None	None	High (main cause of occlusion)	Prevented by covering
Risk of sludge	High (main cause of occlusion)	Moderate (reduced by above-papilla)	Low–Moderate (less than PS, but possible with debris)	Moderate (sludge-related occlusion possible)
Risk of migration	Moderate	Moderate	Low	Moderate–High (migration more frequent)
Side-branch occlusion	Low	Low	Low (mesh preserves branches)	Moderate (reduced risk with slim 6 mm design)
Cost-effectiveness	Low (frequent exchanges needed)	Moderate (fewer exchanges than across-the-papilla PS)	High (fewer reinterventions despite higher cost)	Moderate (device cost but fewer failures)
Availability	Widely available worldwide	Available, but dedicated devices limited	Widely available worldwide	Limited availability (still investigational in many regions)
Evidence strength	High (long history, RCTs available)	Moderate (recent RCTs, promising)	High (multiple RCTs, guideline-recommended standard)	Low (early RCT + retrospective)

PS, plastic stent; MS, metal stent; RCT, randomized controlled trial.
